# Compression or tension? The stress distribution in the proximal femur

**DOI:** 10.1186/1475-925X-5-12

**Published:** 2006-02-20

**Authors:** KE Rudman, RM Aspden, JR Meakin

**Affiliations:** 1 Department of Orthopaedic Surgery, University of Aberdeen, Foresterhill, Aberdeen, AB25 2ZD, UK

## Abstract

**Background:**

Questions regarding the distribution of stress in the proximal human femur have never been adequately resolved. Traditionally, by considering the femur in isolation, it has been believed that the effect of body weight on the projecting neck and head places the superior aspect of the neck in tension. A minority view has proposed that this region is in compression because of muscular forces pulling the femur into the pelvis. Little has been done to study stress distributions in the proximal femur. We hypothesise that under physiological loading the majority of the proximal femur is in compression and that the internal trabecular structure functions as an arch, transferring compressive stresses to the femoral shaft.

**Methods:**

To demonstrate the principle, we have developed a 2D finite element model of the femur in which body weight, a representation of the pelvis, and ligamentous forces were included. The regions of higher trabecular bone density in the proximal femur (the principal trabecular systems) were assigned a higher modulus than the surrounding trabecular bone. Two-legged and one-legged stances, the latter including an abductor force, were investigated.

**Results:**

The inclusion of ligamentous forces in two-legged stance generated compressive stresses in the proximal femur. The increased modulus in areas of greater structural density focuses the stresses through the arch-like internal structure. Including an abductor muscle force in simulated one-legged stance also produced compression, but with a different distribution.

**Conclusion:**

This 2D model shows, in principle, that including ligamentous and muscular forces has the effect of generating compressive stresses across most of the proximal femur. The arch-like trabecular structure transmits the compressive loads to the shaft. The greater strength of bone in compression than in tension is then used to advantage. These results support the hypothesis presented. If correct, a better understanding of the stress distribution in the proximal femur may lead to improvements in prosthetic devices and an appreciation of the effects of various surgical procedures affecting load transmission across the hip.

## Background

**Figure 1:**
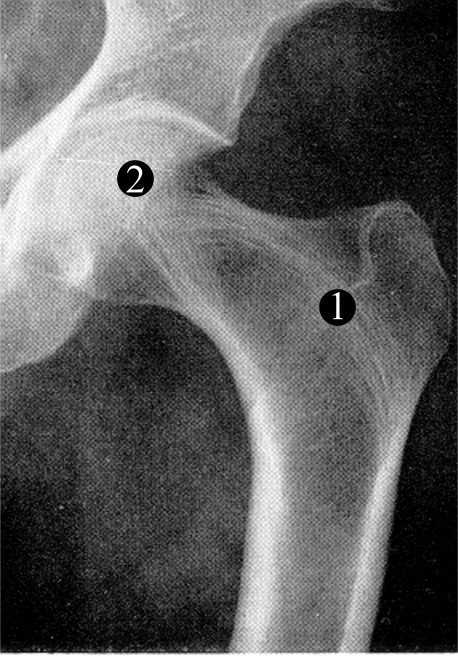
**The human hip joint**. Radiograph of the human proximal femur and acetabulum in which the two main systems of trabeculae (group 1 and 2) are indicated. These are traditionally known as the principle tensile and compressive trabeculae respectively, a questionable nomenclature.

Despite recent advances in modelling, and the success of total hip replacements, it is still not clear what stresses are generated within the proximal femur (the head and neck) during physiological loading. The traditional description is based on the work of the nineteenth century engineer, Cullman, who observed the drawings of the anatomist, Meyer, and likened them to the stress pattern of a crane which he was currently analysing [1]. He proposed that a load representing body weight applied to the femoral head, with the lower end of the femoral shaft fixed, would tend to bend the femoral neck. This would generate tension on the lateral side and compression on the medial side of the femoral shaft. In the proximal femur, this model has led to the group 1 trabeculae in Figure 1 being called the "principal tensile system" and the group 2 trabeculae the "principal compressive system". Consequences of this model are that it predicts a state of zero stress along the front and rear of the femur, a bending moment which must be resisted by the knee joint, and a large downwards deflection of the femoral head [2]. This model of the femur was adapted by Pauwels [3] by including an abductor muscle force to analyse, in the coronal plane, the one-legged stance phase during gait. His analysis reduced, but did not eliminate, tension in the femur. With a few minor variations, this view of how the femur functions still prevails.

However, a small number of dissenters have drawn attention to the number of muscles that cross the articulation of the hip. These muscles apply forces that pull the femoral head into the acetabulum and should not be ignored [4-6]. In addition, a tough, fibrous capsule, within which three distinct ligaments have been described [7,8], encloses the joint. These ligaments are recognized as being thick and strong, and they are prestrained in the upright posture [8,9]. The orientation of their collagen fibres, representing the direction in which they can best resist tensile forces [10,11], is predominantly parallel with the femoral neck. These have never been included in any model of the hip. It has been proposed that the forces due to these muscles and ligaments would result in all the trabeculae being in compression during most normal activities [4,6]. The conflict between this and the traditional model has never been satisfactorily resolved.

Many subsequent studies have investigated the stress distribution within the human femur [2,3,12-16], but most of these have concentrated on stresses developed in the femoral shaft and the effects of implanted devices. Surprisingly little attention has been paid to stress distributions in the natural proximal femur. Most studies assume the traditional model and do not include ligaments, muscles or the acetabulum. In addition, the proximal femur is generally represented as a homogeneous, isotropic solid, with the exception of a recent FE model based on microCT (Computer Tomography) data which modelled the individual trabeculae [16]. This model is impressive but still limited in that it does not include ligaments or muscles and the loading over the femoral head is approximated. Another model started with an isotropic distribution of trabeculae and investigated how adaptation to load might predict the organization of trabeculae; they did not, however, investigate factors affecting the distribution of stress [17,18]. Linear and non-linear material properties were included in a study of hip fracture but the emphasis was on von Mises stresses and failure [19,20], rather than on stress magnitudes and directions.

Given that bone is stronger in compression than tension [21,22] and that there exists a clear trabecular architecture in the adult hip, our hypothesis is that the majority of the head and neck of the femur are in compression and the group 1 trabeculae (Fig. 1) function as an arch-like structure, similar to a flying buttress [23], transferring compressive forces to the shaft during normal activities. The group 2 trabeculae (Fig. 1) will transmit forces both directly to the medial aspect of the shaft and partly to the arch of the group 1 trabeculae. It is proposed that the horizontal compressive forces required to provide the abutments in the femur are generated on the lateral side by the ligaments and muscles connecting the femur to the pelvis and on the medial aspect by the acetabulum. The aim of this study was a proof of principle to determine whether reasonable boundary conditions could be found to support this hypothesis. In order not to introduce excessive complexity and to explore the conditions under which different stress distributions might arise, we have developed first a 2D FE model. In this model we included representations of the capsular ligaments, in which we could vary the forces holding the femoral head into the acetabulum. We also included regions of trabecular bone with an increased modulus to represent the greater structural density arising from the internal architecture. Initially, we investigated two-legged stance where the only loads are those due to body weight and the ligaments of the hip. No muscles were included since electromyography has shown that there is very little muscle activity during two-legged stance [24]. We then modelled the one-legged stance of Pauwels by including an abductor force [3]. Further muscles were not included because of the difficulties of accurately representing them in a 2D model.

## Methods

**Figure 2:**
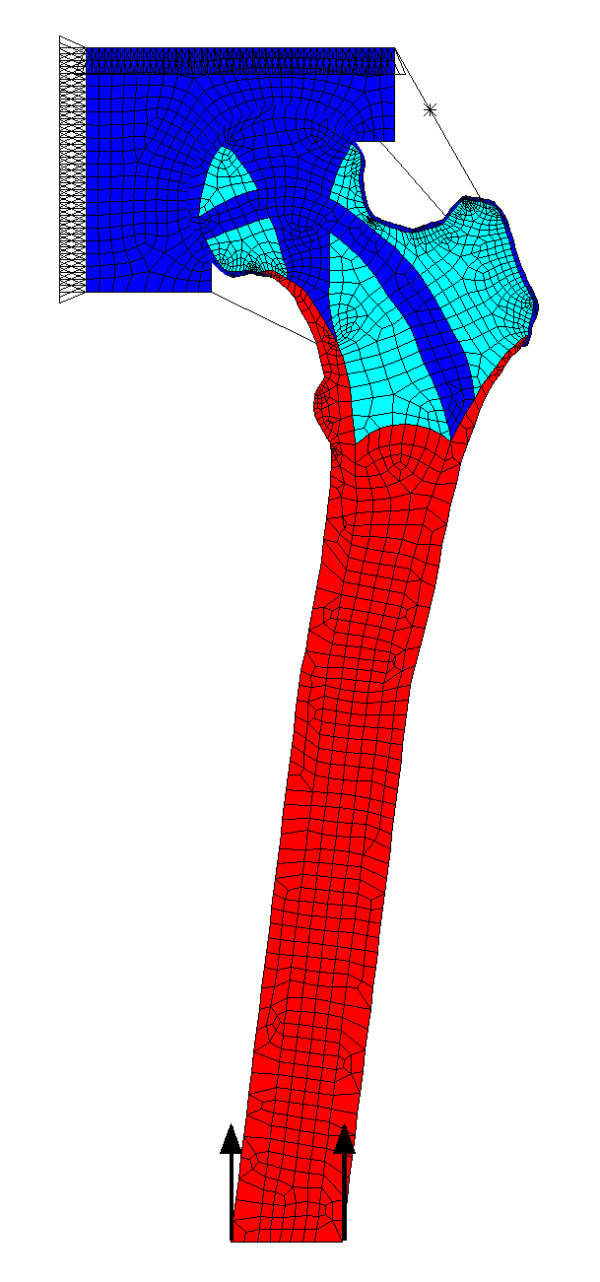
**Finite element model of the hip**. Finite element model of the proximal femur and acetabulum. Spring elements join the two components and represent the capsular ligaments. The model is fixed at the top and loaded through the base of the femur. Also shown with an asterisk is the link-element used to apply the abductor force in the model representing one-legged stance. Red: modulus 17 GPa Blue: modulus 400 MPa Turquoise: modulus 100 MPa.

An FE model was constructed comprising the shaft and proximal femur articulating freely with a representation of the acetabulum (Figure 2). The acetabulum was deemed to be fixed and loads were applied through the distal femur. The geometry of the proximal femur was taken as the average shape derived from a series of anterior-posterior radiographs of healthy women [25]. Cortical bone in the shaft was assigned a Young's modulus of 17 GPa [12,13]. The trabecular bone in the head and neck was assigned Young's modulus values that reflected the structural organisation within the femur. The group 1 and group 2 trabeculae (Figure 1) were given a modulus of 400 MPa while the surrounding trabecular bone had a modulus of 100 MPa [26] (Figure 2). The acetabulum was also assigned a modulus of 400 MPa. All were given a Poisson's Ratio of 0.33 [12,27]. The lattice work structure of the trabeculae was ignored, allowing the material to be modelled as a homogeneous, isotropic solid at the microscopic scale; common practice when investigating bone stress [17,18]. The femoral shaft was oriented at 7° to the vertical [7] (Figure 2). This angle, which is at the lower end of the normal range, represents a 'worst case' scanario for our model, since larger angles would make the group 1 trabeculae more vertical and tend to favour our hypothesis. Having chosen the angle at which the femur was inclined, the length of the shaft was set such that its distal end was vertically below the centre of the femoral head. This was done to fulfill the condition that there should be a zero moment about the femoral head, and at the knee, when body weight was applied.

The FE model was developed using ANSYS 8.0 software (ANSYS, Inc., USA). The model consisted of 2162 eight-node quadrilateral elements (PLANE82) (Figure 2) using the plane stress option (no thickness option was selected). Contact between the femoral head and acetabulum was modelled by the use of contact elements (CONTA172) and target elements (TARGE169), which allowed the femur to move freely inside the acetabulum. The co-efficient of friction used was effectively zero, to ensure that only normal forces, not shear, were transmitted across the contact region. Capsular ligament forces were represented by tension only link elements (LINK10) acting as springs with a spring constant of 127 N mm^-1 ^[8] (Figure 2). A prestrain was applied to them to represent the initial tension present in the ligaments [28]. For analysis of two-legged standing, a force of 300 N was applied as a distributed load to the distal femur while the acetabulum was constrained in the vertical and horizontal directions. The model was solved first with no ligaments, to represent the traditional model. Then the link elements were included with initial prestrains of 2.5%, 5%, and 10%. These strains are not necessarily those the ligaments would experience *in vivo*, but are the strains required in order that the link elements apply forces that would be expected to include those experienced *in vivo*. Because the femoral head was allowed rotational freedom inside the acetabulum, the position of the femur could be adjusted automatically during the solving process until equilibrium, and a solution, was achieved. The final ligamentous forces were always less than the failure load of the capsule reported in the literature [8]. Joint reaction forces were found by summing the nodal forces over the surface of the femoral head.

To analyse one-legged stance, an abductor force was included as described by Pauwels in his free-body model [3]. This model was chosen as being one of the pioneering, and now standard, analyses with which many studies are compared. To represent this model, the shaft of the femur had to be extended to simulate loading slightly contralateral to the midline of the body. Then, body weight (minus one leg) of 600 N was applied at the base of the shaft, a horizontal distance of 112 mm from the centre of the femoral head. The abductor force used by Pauwels of 1764 N at an angle of 30° [3] was applied between the greater trochanter and the representation of the pelvis. This force was modelled using a pre-tension element (PRETEN66). The capsule was represented, as above, using link elements and a prestrain of 5 % was chosen as being in the mid-range of those used above.

## Results

### Traditional model

**Figure 3:**
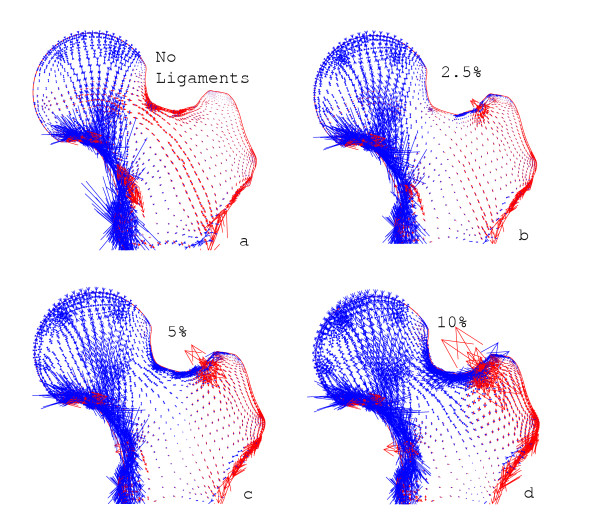
**Two-legged stance**. The distribution of principal stresses as the tension in the spring elements is increased. The length and direction of the arrows show the relative magnitude and direction of compression, in blue, and tension, in red. (a) Without spring elements, showing generation of tension in the group 1 trabeculae. Increasing the initial strain to (b) 2.5%, (c) 5% and (d) 10% shows how the stresses become compressive throughout the proximal femur and focussed into the arch of the group 1 trabeculae.

The results were displayed as plots of principal stress to show the relative magnitude and direction of the internal stresses. In the absence of the link elements representing the capsular ligaments, the stresses in the proximal femur (Figure 3a) followed a similar pattern to those predicted by the traditional model. Compressive stresses transmitted force into the medial shaft through the group 2 trabeculae, and tension was generated across most of the central and lateral areas of the proximal femur, especially in the group 1 trabeculae.

### Two-legged stance

When capsular forces were included, a region of compressive stress, which grew larger as the prestrains were increased, extended over most of the proximal femur apart from close to the attachments of the link elements (Figure 3b–d). Between 2.5% (Figure 3b) and 5% (Figure 3c) prestrain, the stresses in the proximal femur became primarily compressive. Total ligament forces in these cases were 120 N and 238 N. As with the traditional model, compressive stress was transmitted through the group 2 trabeculae into the medial shaft. The group 1 trabeculae now also transmit compressive stress along their arch-like arrangement towards the lateral shaft. With a ligament strain of 5%, the joint reaction force was calculated to be 476 N at an angle of 24° to the vertical. As the prestrain was increased further, to 10% (Figure 3d), the compressive stresses became larger in magnitude but maintained the same direction and focus along the trabecular systems as found with 5% prestrain. The total ligamentous force was now 475 N. The absolute magnitudes of the internal stresses mean little, as this is only a 2D model, but the patterns of stress were consistent. The high tension in the lateral cortex is a consequence of the point forces due to the ligamentous attachments in this 2D model.

### One-legged stance

**Figure 4:**
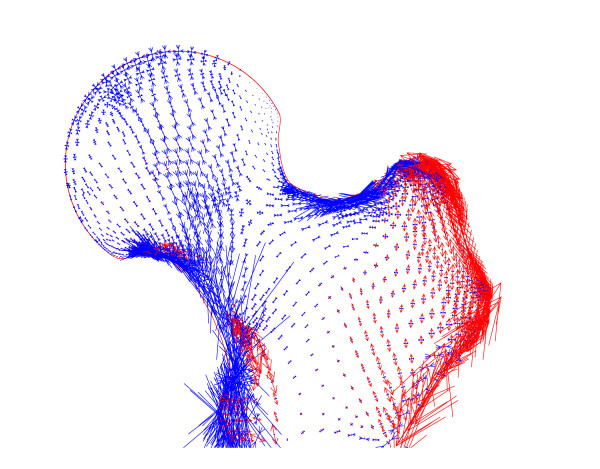
**One-legged stance**. The distribution of principal stresses in a model of one-legged stance with spring elements at a prestrain of 5%, a vertical force of body weight applied to the femur and an abductor force of three times body weight. The lengths and directions of the arrows show the relative magnitudes and directions of compression, in blue, and tension, in red.

When an abductor force was included, and the configuration changed to that of one-legged stance, the state of stress was still found to be compressive throughout the proximal femur (Figure 4). The directions of the compressive stresses were now different from those for two-legged stance, because of the large abductor muscle force. The magnitude of this force, and the fact that it was applied at a single node, were also the source of the large tensions generated in the lateral cortex. The joint reaction force was found to be 2520 N at an angle of 23° to the vertical and the total force due to the ligaments was 227 N.

## Discussion

The purpose of this study was to begin to test the hypothesis that the proximal femur functions primarily in compression, not bending-induced tension as is widely believed. Given the dearth of studies of the internal stress distribution in the proximal femur, it was intended as a pilot study and a proof of principle. If no compression could have been generated in this model with any sensible boundary conditions there would be little point continuing to a full 3D model and the complexity of representing all the muscle and ligamentous attachments that will require. To be accurate, it is necessary to model as closely as possible all the forces applied to the system; it is not sufficient to apply a joint reaction force because the local stress distribution is strongly dependent on the local forces. Clearly, a 2D model is limited in this respect, but it gives an indication of the forces that need to be included, ligaments as well as muscles, and shows the need to try to represent the internal architecture and the pelvis.

Loading the isolated femur through the femoral head while the base is fixed generates tension in the group 1 trabeculae, as found in the traditional model. Similar results were obtained when the acetabulum was included and loads applied through the femur in the absence of ligaments. Validation is difficult but the closeness of the stress distribution to that traditionally derived is one indicator of the validity of the model. There is also reasonable agreement with published values of joint reaction forces measured using instrumented prostheses as described below. The effects of different element sizes were explored and found to have little effect on the stress distribution.

In our model, including a representation of the joint capsule changed the internal stresses from tensile to compressive. The plots of principal stresses show the generation of primarily compressive forces in the directions we hypothesize, in contrast to the representation of the traditional model. This was true for all ligament forces used and for both two-legged and one-legged stance. The extent of the region of compression increased as the forces increased, although we have not attempted to quantify this because of the inherent limitations of trying to represent a solid object in two dimensions. Now that we have demonstrated that there is a substantial amount of compression in the proximal femur, we can develop a 3D model and produce more reliable quantitative results. The range of ligament forces used shows the gradual development of regions of compression and the corresponding shrinking of regions of tensile stress as ligament forces increase.

The joint reaction forces predicted in both models are in reasonable agreement with the magnitudes of those measured using instrumented prostheses. We calculated a joint reaction force of 0.68 body weight (BW) (476 N) in two-legged stance with a link element strain of 5%. This compares with between 0.59–1.0 BW for two-legged stance measured by Bergmann et al. [29], and either 400–500 N [30] or 0.9–1.3 BW [31] in separate studies reported by Taylor et al. For one-legged stance, our calculated joint reaction force was 3.6 BW at 23° to the vertical, compared with Pauwels' calculated value of 4 BW at 16° [3]. Measured joint reaction forces have been 1.8–2.3 BW [31] and values of between 2.2 and 3.7 BW that can be calculated in the frontal plane from Bergmann's data [29], both reasonably close to our model predictions. The predicted angles of those forces, however, are slightly different. These differences are not surprising, as not only is our model 2D, but we have included ligaments, thereby adding a horizontal component to the force. This component will not be present in studies using instrumented prostheses because the ligaments are almost invariably cut during surgery.

Investigating the one-legged stance analysed by Pauwels [3] suggested that compression was still the predominant stress in the head and neck of the femur but, in this model, the largest stress was no longer oriented along the arch of the group 1 trabeculae. This model, however, was adapted from that developed by Pauwels to investigate joint reaction forces using a free-body analysis. The simplified forces and constraints severely limit the conclusions that can be drawn from a stress analysis. We anticipate that distributed muscle forces and additional active muscles will considerably affect the calculated stress distribution. A more detailed 3D model is being developed to explore this more thoroughly.

The hip, despite its apparent simplicity, is surrounded by a very complicated arrangement of muscles and ligaments. The joint capsule and its ligaments are recognized as being among the thickest and strongest ligaments of the body and they are always in tension to prevent dislocation of the hip; a rare event [7,8]. They also contribute to the stability of the standing posture by counterbalancing the weight of the torso [32]. These tissues have been ignored in previous models and yet, anatomically, represent some substantial elastic structures. The muscles include some of the most powerful in the body and the orientations of the muscle fibres, especially in the deeper muscles, suggest that they exert forces with a large component pulling the femoral head into the acetabulum [7]. Thus, when they are active, they will also contribute to the compression applied to the femoral head. In addition, few FE studies have tried to represent the internal architecture of the trabeculae. For simplicity, we first modelled the femur with uniform properties (data not shown) and still the stresses were predominantly compressive. Increasing the modulus in the region of the group 1 trabeculae, to represent their increased density, changed the relative magnitudes of the stresses, though without changing their sign.

## Conclusion

The results of our model demonstrate that when ligamentous and muscular forces are included, the stresses in proximal femur are predominantly compressive. This would appear to make better use of the mechanical properties of bone, which is stronger in compression than in tension [33]. Combining those properties with the arch-like structure of the trabeculae provides a powerful means of transmitting forces from the femoral head in to the shaft without generating large bending moments in the neck [23,34]. To calculate the stresses in the proximal femur, all the locally applied forces have to be included; it is not sufficient to use a resultant force. A proper understanding of the stress distribution in the femur would be expected to have implications for surgery and implant design.

## Competing interests

The author(s) declare that they have no competing interests.

## Authors' contributions

KER performed the FE analysis, participated in the design and planning of the project and wrote the first draft of the manuscript. JMR participated in the design and planning of the project, supplied the detailed technical expertise in FE modelling and helped to draft and revise the manuscript. RMA conceived the study, participated in its design and coordination and helped to draft and revise the manuscript. All authors read and approved the final manuscript.
